# Development and validation of a multiplex qPCR assay for detection and relative quantification of HPV16 and HPV18 *E6* and *E7* oncogenes

**DOI:** 10.1038/s41598-021-83489-2

**Published:** 2021-02-17

**Authors:** Alexia Bordigoni, Anne Motte, Hervé Tissot-Dupont, Philippe Colson, Christelle Desnues

**Affiliations:** 1 Aix-Marseille Université, IRD 198, Assistance-Publique des Hôpitaux de Marseille, UMR Microbes, Evolution, Phylogeny and Infections (MEPHI), Marseille, France; 2 Aix-Marseille Université, Université de Toulon, CNRS, IRD, Mediterranean Institute of Oceanography (MIO), MEB, UM 110, 163 avenue de Luminy, Case 901, Bâtiment OCEANOMED-Méditerranée, 13288 Marseille Cedex 09, France; 3 IHU Méditerranée Infection, Marseille, France

**Keywords:** Human papilloma virus, PCR-based techniques

## Abstract

Human papillomaviruses (HPV) play a key role in promoting human anogenital cancers. Current high-risk HPV screening or diagnosis tests involve cytological or molecular techniques mostly based on qualitative HPV DNA detection. Here, we describe the development of a rapid quantitative polymerase chain reaction (qPCR) detection test of HPV16 and HPV18 oncogenes (*E6* and *E7*) normalized on human gene encoding *GAPDH*. Optimized qPCR parameters were defined, and analytical specificities were validated. The limit of detection was 10^1^ for all genes tested. Assay performances were evaluated on clinical samples (n = 96). Concordance between the Xpert HPV assay and the triplex assay developed here was 93.44% for HPV16 and 73.58% for HPV18. HPV co-infections were detected in 15 samples. The systems developed in the present study can be used in complement to traditional HPV tests for specifically validating the presence of HPV16 and/or HPV18. It can also be used for the follow-up of patients with confirmed infection and at risk of developing lesions, through the quantification of *E6* and *E7* oncogene expression (mRNA) normalized on the *GAPDH* expression levels.

## Introduction

Human papillomaviruses (HPVs) are non-enveloped icosahedral particles with a size of 50–60 nm diameter^1^. These circular double-stranded DNA viruses have a genome size close to 8 kilobases that includes open reading frames (ORF) encoding E1–E7 proteins involved in the transactivation of transcription, transformation, replication and viral adaptation into the host cells^2,3^. HPVs are involved in the development of cutaneous or genital warts (or condyloma) and of benign or malignant tumors^4^. Currently, more than 200 HPV types (https://pave.niaid.nih.gov/) are described and categorized in two groups, namely low-risk (LR) and high-risk (HR) HPVs according to their capacity to promote cell transformation that leads to cancer development. According to the Center for Disease Control and prevention, more than 90% of anal and cervical cancers and 50% of vaginal, vulvar, and penile cancers are due to HPVs^5^. HPV16 and 18 are frequently associated with anal cancer (87%), cervical, vulvar or penile cancer (70%), vaginal cancer (64%), but also head and neck cancer (85%)^6^.

HPVs infect basal epithelial cells of the skin (cutaneotropic) and of the mucosa of the anogenital and the oropharyngeal tracts (mucosotropic). Cancerous lesions are due to persistence of HPVs that are not cleared in the epithelium by the human immune system^4,7,8^. Indeed, a productive viral replication is associated with a low-grade intraepithelial lesion^9^. The progression to precancerous and cancerous lesion up to cervical intraepithelial neoplasia and eventually to cervical cancer occurs with a persistent HR-HPV infection and the integration of the HPV genome into the host chromosome mediated by host DNA replication and recombination^10^, the loss or disruption of E2 protein and the subsequent upregulation of *E6* and *E7* oncogenes expressions^11^. These oncoproteins are involved in malignant transformation by altering cell cycle regulation and telomere maintenance via the telomerase activation and the degradation of the tumor suppressors p53 by E6 and retinoblastoma protein (Rb) by E7^12–14^. In contrast, LR-HPV oncogenes are less able to interfere with p53 and Rb functions than E6/E7 proteins from HR-types^12,15^.

Nowadays, HR-HPV screening or diagnosis mostly involve cytological techniques that have lowest specificity and sensitivity than molecular techniques^16,17^ but detection of HPV DNA is becoming the first line strategy for cervical cancer in women older than 30 years^18–20^. Commercially available molecular tests are mainly based on the detection of HPV DNA such as Hybrid Capture 2 assay (QIAGEN)^21^, Cervista HPV HR^22^ or HPV 16/18^23^ (HOLOGIC) Care HPV (QIAGEN)^24^ and Cobas HPV (ROCHE DIAGNOSTICS)^25^, are mainly based on the detection of HPV DNA. These allow detecting the presence of the virus but not evaluating its oncogenic activity. In cervical infections HPV load and its variations have been shown to predict the persistence and progression of HPV infections and the severity of the lesions^26,27^. In 2010, a study showed that mRNA expression levels of *E6* and *E7* oncogenes were correlated with the severity of cervical lesions^28^, but so far, only few HPV tests target viral mRNAs. They are qualitative (e.g. HPV-Proofer (PRETECT))^29^ and do not discriminate among HPV types (Aptima HPV Assay (HOLOGIC))^30^.

The main aim of the present study was to develop a rapid quantitative detection test of HPV16 and HPV18 infections by measuring *E6* and *E7* oncogene DNA levels normalized by the cellular *GAPDH* gene level. Multiplex detection of these three genes in a single assay enables reduction in cost, time and labor as compared to separate detection methods. This technique allows for an extra support of traditional HPV tests to validate and quantify specifically the presence of HPV16 and/or HPV18 infection. Once persistent infection is confirmed, it could be used for a follow-up of patients at risk of cervical cancer, by correlating the activation of oncogenes to the severity of the lesions.

## Materials and methods

### Primer and probe design

Target sequences of *E6* and *E7* genes were obtained using the NCBI reference genomes of HPV16 (accession number: NC_001526.4) and HPV18 (accession number: X05015.1). The primers and probes were designed to amplify and detect specifically *E6* and *E7* oncogenes of HPV16 and HPV18 using PrimerQuest Tool software with default parameters. The specificity of the primers and probes was checked using the online NCBI BLASTn tool against the human genome (https://blast.ncbi.nlm.nih.gov/). Human GAPDH primers were obtained from previous study^31^.

The hydrolysis probes of *E6*, *E7* and *GAPDH* were labeled on the 5′-end with FAM, VIC and CY5 fluorescent dyes, respectively, and on the 3′-end with a TAMRA compatible quencher for *E6* and *E7* and BHQ (Black Hole Quencher) for *GAPDH*.

The sequences of primers and probes, nucleotide positions on HPV16 or HPV18 reference genomes and amplicon lengths are presented in Table 1. Primers and probes were manufactured by EUROGENTEC (Angers, France).

**Table 1:** Primers and probes used in qPCR.

Target	Oligonucleotide	Sequence 5′–3′	Amplicon length (bp)	Reference	Oligonucleotide position on reference genome	
					From	To
**HPV 16**						
*E6* gene	Primer E6 F	5′-AATGTTTCAGGACCCACAGG-3′	107 bp	This study on HPV16 reference genome NC_001526.4 NCBI	7145	7164
	Primer E6 R	5′-GTTGCTTGCAGTACACACATTC-3′			7230	7251
	Probe E6	5′-ACCACAGTTATGCACAGAGCTGCA-3′			7181	7204
*E7* gene	Primer E7 F	5′-TCAGAGGAGGAGGATGAAATAGA-3′	111 bp		7697	7719
	Primer E7 R	5′-GCACAACCGAAGCGTAGA-3′			7790	7807
	Probe E7	5′-AGAACCGGACAGAGCCCATTACAA-3′			7738	7761
**HPV18**						
*E6* gene	Primer E6 F	5′-ACCCTACAAGCTACCTGATCT-3′	100 pb	This study on HPV18 reference genome X05015.1 NCBI	134	154
	Primer E6 R	5′-ACCTCTGTAAGTTCCAATACTGTC-3′			212	235
	Probe E6	5′-ACGGAACTGAACACTTCACTGCAAGA-3′			159	184
*E7* gene	Primer E7 F	5′-AATTCCGGTTGACCTTCTATGT-3′	102 bp		649	670
	Primer E7 R	5′-GGCTGGTAAATGTTGATGAT-3′			723	742
	Probe E7	5′-TAAGCGACTCAGAGGAAGAAA-3′			681	701
GAPDH	Primer F	5′-GGACCTGACCTGCCGTCTAG-3′	99 pb	He et al. (2017)^31^		
	Primer R	5′-TAGCCCAGGATGCCCTTGAG-3′				
	Probe	5′-CCTCCGACGCCTGCTTCACCACCT-3′				
HPV universal	MY09	5′-GCMCAGGGWCATAATAATGG-3′	450 pb	Cossellu et al. (2018)^34^		
	MY11	5′-CGTCCMARRGGAWACTGATC-3′				

### Positive control synthesis for quantification of gene copy number

HPV DNAs were extracted using a BioRobot EZ1 instrument (QIAGEN) from ATCC HeLa cells for HPV18 (CCL-2) and ATCC SiHa cells for HPV16 (HTB-35) cultured in a MEM medium (GIBCO) supplemented with 10% fetal bovine serum (GIBCO), 1% non-essential amino acids 100X (GIBCO), 1% glutamine 100X (GIBCO), 1% sodium pyruvate 100X (GIBCO) at 37 °C with 5% CO_2_. PCRs were performed according to the manufacturer recommendations with AmpliTaq Gold 360 Master Mix kit (THERMOFISCHER) in a 25 µL volume containing 12.5 µL of 2X AmpliTaq Gold Mix, 0.5 µL of each primer at 10 µM and 2 µL of template DNA.

Amplicons were produced by standard PCR in a Mastercycler nexus gradient (EPPENDORF) according to the following program: 10 min at 95 °C; then 40 cycles of 30 s at 95 °C, 30 s at 60 °C, and 20 s at 72 °C; and a final extension step of 7 min at 72 °C. PCR products were analyzed by electrophoresis on a 2% agarose gel and purified by QIAquick PCR purification (QIAGEN) before being cloned into a PGEM-T easy vector (PROMEGA). The ligation reaction was conducted overnight at 4 °C according to the manufacturer recommendations. JM109 competent cells (PROMEGA) were transformed by heat shock, before being spread with beads onto Luria–Bertani (LB) (BD DIFCO) agar plates containing 100 µg/ml ampicillin (SIGMA), 0.5 mM isopropyl-β-d-thiogalactopyranoside (EUROMEDEX) and 80 µg/ml X-gal (EUROMEDEX). Cells were incubated at 37 °C for 24 h or during the weekend at room temperature. Transformants were analyzed by PCR using SP6 (5′-TATTTAGGTGACACTATAG-3′) and T7 primers (5′-TAATACGACTCACTATAGGG-3′) according to the following program: 10 min at 95 °C; then 40 cycles of 30 s at 95 °C, 30 s at 55 °C, and 20 s at 72 °C; and a final extension step of 7 min at 72 °C. Positive *E6* and *E7* clones for each HPV genotype and *GAPDH* clones were placed into 7 mL of LB broth containing 100 µg/mL of ampicillin then incubated overnight at 37 °C under shaking (~ 200 rpm). Plasmid DNA was extracted using the Smart Pure Plasmid Kit (EUROGENTEC). Purified plasmids were linearized by digestion with the *NcoI* restriction enzyme during 15 min at 37 °C according to the manufacturer’s instructions. Digestion products were run on a 1% agarose gel and the band was purified using the QIAquick Gel Extraction (QIAGEN). The plasmid concentration was quantified by Nanodrop 2000 (THERMO SCIENTIFIC) for HPV16 and by Multiskan GO (THERMO SCIENTIFIC) for HPV18. The copy numbers (copies/µL) were determined with the following formula:

$$ Copy\;numbers\left( {{\text{copies/}}{\upmu} {\text{l}}} \right) = \frac{{Q\left( {{\text{ng}}} \right) \times {\mathcal{N}}_{{A \left( {Da} \right)}} }}{{L\left( {{\text{pb}}} \right) \times 10^{9}  \times M\left( {{\text{g/mol}}} \right)}} $$

Plasmid copy numbers were 1.59 × 10^10^ copies/µL (*GAPDH*), 1.44 × 10^10^ copies/µL (HPV16, *E6),* 9.9 × 10^9^ copies/µL (HPV16, *E7*), 1.54 × 10^10^ copies/µL (HPV18, *E6*) and 1.58 × 10^10^ copies/µL (HPV18, *E7*). Serial dilutions (from 10^7^ to 10^0^) of the control plasmids were aliquoted and stored at − 20 °C until further use. All steps of qPCR development were run with 2 µL of plasmid solution.

### Multiplex qPCR development

Optimization of HPV16 and HPV18 multiplex was run on a CFX C1000 Touch (BIORAD) to determine the most appropriate annealing temperature (54 °C, 56.4 °C, 57.8 °C, 60 °C), primer concentration (400 nM, 600 nM, 800 nM, 1000 nM) and probe concentration (50 nM, 100 nM, 150 nM, 200 nM) for each gene. According to the manufacturer protocol, qPCRs were performed with a LightCycler 480 Probes Master (ROCHE) kit in 20 µL containing primers and probes (at different concentrations listed above), 10 µL 2X LightCycler 480 probe Master mix, 0.5 µL Uracil-DNA Glycosylase (1 U/µL, THERMO SCIENTIFIC) and 2 µL of DNA template. Multiplex assays were performed in biological and technical triplicates on 10^5^ plasmid copies and using the following steps: 2 min at 50 °C; 5 min at 95 °C; 40 cycles of 10 s at 95 °C; 20 s at Tm; and a final 1 s at 72 °C.

### Specificity of the primers

The specificities of the primers-probe couples were tested on each plasmid produced as mentioned above at the concentration of 10^5^ copies in a multiplex qPCR by using the optimized parameters on a CFX C1000 Touch (BIORAD). Each run consisted in three steps: 2 min at 50 °C, 5 min at 95 °C, then 40 cycles of 10 s at 95 °C and 20 s at 60 °C.

### Efficiency and sensitivity of the PCR systems

PCR efficiency and sensitivity of each primers-probe couple with the optimized conditions were determined using a quantitative standard curve (serial dilution of the positive control plasmid from 10^7^ to 10^0^ copies). All assays were performed in biological and technical triplicates on a CFX C1000 Touch (BIORAD) with the following program: 2 min at 50 °C; 5 min at 95 °C; 40 cycles of 10 s at 95 °C and 20 s at 60 °C. Assays were validated with a slope of regression curve between − 3.9 and − 2.9 cycles/log_10_, a R^2^ > 0.98 and an efficiency range between 80 and 120%^32^. The limit of detection (LOD) of the assays was defined as the lowest concentration (target gene copy number per reaction) for which ≥ 95% of test runs gave positive results.

### Multiplex reproducibility

Intra- and inter-assay reproducibility was evaluated based on the cycle threshold (Ct), and the coefficient of variation (CV) of both the biological and the technical replicates, using a concentration of 10^5^ copies of each target gene.

### Triplex validation and application on clinical samples

As reference for HPV detection, we used the diagnostic results obtained at the IHU Méditerranée Infection laboratory using the Xpert HPV Assay (CEPHEID) from 96 clinical materials (67 cervico-vaginal smears, four vaginal swabs, one cervix, 19 anal smears, five rectal swabs) collected in liquid-based media (PreservCyt, HOLOGIC) (Supplementary Table 1). The Xpert HPV Assay is a real-time polymerase chain reaction assay using disposable cartridges that is able to detect 14 types of high-risk HPV DNA (types 16, 18, 31, 33, 35, 39, 45, 51, 52, 56, 58, 59, 66, 68). Partial genotyping is provided for HPV16, HPV18 and/or 45, and HR-HPV other than 16, 18 or 45. The 96 clinical samples consisted in 43 HPV16 positive, 35 HPV18/45 positive, 3 HPV16 and HPV18/45 positive, seven positives for other HR-HPVs and 8 HPV negative.

For testing the multiplex systems developed in the present work, DNA extraction from 1 mL of each of the 96 original clinical samples was performed using the BioRobot EZ1 instrument (QIAGEN) or High Pure Nucleic Acid Large Volume extraction kits (ROCHE) according to the manufacturer recommendations. qPCR on DNA samples (5 µL) were run on a CFX C1000 Touch (BIORAD) according to the optimized parameters determined in this work. qPCR products with Ct values > 38 and PCR products visible on a 2% agarose gel using universal HPV primers were verified by Sanger sequencing using Big Dye Terminator v1.1 Cycle Sequencing Kit (LIFE TECHNOLOGIES) according to manufacturer recommendations on a 3500XL Genetic Analyzer capillary sequencer (THERMOFISHER).

Using the standard curve, the copy number of each HPV18/16 oncogenes and *GAPDH* was determined. The HPV viral load was calculated according to the following formula^26^:

$$ Viral\;load\left( {HPV\;oncogene\frac{copies}{{10^{6} \;cells}}} \right) = \frac{number\;of\;HPV\;oncogene\;copies}{{number\;of\;GAPDH\;copies{/}2}} \times 10^{6} $$

### Ethics statement

Clinical samples used in this study have been collected in the setting of routine standard clinical management and informed consent was obtained from all subjects. The study was approved by the ethical committee of the University Hospital Institute Méditerranée Infection (No. 2020-03). The study was performed according to the good clinical practices recommended by the Declaration of Helsinki (and its amendments) and all methods were carried out in accordance with relevant guidelines and regulations.

### Statistical analyses

All statistical analyses were performed using the Prism 7 software (GRAPHPAD). Two-way ANOVA and Tukey’s multiple comparison test were performed to analyze the differences between the Ct and RFU values (for primer and probe concentrations) between conditions, with H0 rejected for a p-value < 0.05. Concordance and Cohen’s kappa value^33^ were calculated to determine the level of agreement of HPV16 and HPV18 detection results between the multiplex systems developed in this study and the Xpert HPV Assay. Kappa values of < 0.20, 0.21–0.40, 0.41–0.60, 0.61–0.80 and 0.81–1.00 were considered poor, fair, moderate, good and very good agreement, respectively.

## Results

### Optimization of the multiplex qPCR parameters

To optimize the fluorescence rate of each gene in the multiplex system, four primer concentrations (400 nM, 600 nM, 800 nM, 1000 nM) and four probe concentrations (50 nM, 100 nM, 150 nM, 200 nM) were tested. The optimal concentration for the two multiplex qPCRs (HPV16 or HPV18) was determined as the highest relative fluorescence unit (RFU) for the lowest Ct value (Table 2a,b). Results showed that best RFU means were obtained with 200 nM of probe (p ≤ 0.05) and that there were no significant differences between Ct values and primer concentrations independently of probe concentration (p > 0.05). The lowest concentration of primers (400 nM) was then selected as optimal condition.

**Table 2:** Intra- and inter-assay reproducibility of HPV16 (a) and HPV18 (b) qPCR multiplexes for selected primer and probe concentrations.

Probe concentrations	Primer concentrations	E6				E7				GAPDH			
		Coefficient of variation				Coefficient of variation				Coefficient of variation			
		Ct mean^a^	RFUs mean	Intra-assay	Inter-assay (%)	Ct mean^a^	RFUs mean	Intra-assay	Inter-assay (%)	Ct mean^a^	RFUs mean	Intra-assay	Inter-assay (%)
**(a) HPV16**													
50 nM	400 nM	24.13	809	0.05% ≤ CV ≤ 1.7%	0.24	23.31	417	0.94% ≤ CV ≤ 1.81%	0.84	22.05	265	0.47% ≤ CV ≤ 1.82%	1.30
	600 nM	24.35	826	0.35% ≤ CV ≤ 0.92%	0.91	23.46	427	0.88% ≤ CV ≤ 1.70%	0.54	22.41	257	0.00% ≤ CV ≤ 0.81%	0.32
	800 nM	24.44	823	0.83% ≤ CV ≤ 0.97%	0.70	23.42	433	1.34% ≤ CV ≤ 3.34%	1.39	22.47	260	0.55% ≤ CV ≤ 1.36%	1.15
	1000 nM	24.41	823	0.24% ≤ CV ≤ 0.42%	0.81	23.52	430	0.39% ≤ CV ≤ 3.17%	0.63	22.48	254	0.27% ≤ CV ≤ 0.52%	0.08
100 nM	400 nM	24.11	1892	0.52% ≤ CV ≤ 3.30%	0.80	23.35	951	0.35% ≤ CV ≤ 7.38%	1.90	22.64	616	1.45% ≤ CV ≤ 2.89%	0.83
	600 nM	24.27	1770	0.18% ≤ CV ≤ 0.41%	0.45	23.06	945	0.98% ≤ CV ≤ 2.21%	0.15	22.54	582	0.40% ≤ CV ≤ 0.58%	0.81
	800 nM	24.35	1726	0.21% ≤ CV ≤ 0.78%	0.75	23.00	937	0.91% ≤ CV ≤ 1.29%	1.84	22.44	564	0.59% ≤ CV ≤ 2.37%	1.63
	1000 nM	24.33	1731	0.24% ≤ CV ≤ 0.84%	0.55	23.02	927	1.11% ≤ CV ≤ 1.54%	0.06	22.51	555	0.34% ≤ CV ≤ 1.53%	1.04
150 nM	400 nM	24.19	2435	0.10% ≤ CV ≤ 0.42%	0.11	23.23	1311	1.58% ≤ CV ≤ 1.79%	1.63	22.50	827	0.54% ≤ CV ≤ 0.90%	1.20
	600 nM	24.19	2465	0.25% ≤ CV ≤ 0.66%	0.26	23.23	1316	0.26% ≤ CV ≤ 2.98%	1.41	22.58	800	0.52% ≤ CV ≤ 0.81%	1.18
	800 nM	24.37	2496	0.47% ≤ CV ≤ 1.30%	0.17	23.35	1340	0.86% ≤ CV ≤ 1.60%	1.58	22.43	807	0.41% ≤ CV ≤ 1.62%	2.90
	1000 nM	24.28	2508	0.17% ≤ CV ≤ 0.31%	0.90	23.39	1341	0.17% ≤ CV ≤ 1.79%	1.99	22.63	799	0.15% ≤ CV ≤ 0.59%	1.51
200 nM	400 nM	24.29	3279	0.12% ≤ CV ≤ 0.94%	0.76	23.30	1747	0.25% ≤ CV ≤ 2.07%	0.63	22.93	1143	0.78% ≤ CV ≤ 1.14%	1.19
	600 nM	24.35	3235	0.04% ≤ CV ≤ 0.62%	0.43	23.45	1694	0.42% ≤ CV ≤ 4.02%	0.90	22.79	1079	0.48% ≤ CV ≤ 1.67%	0.86
	800 nM	24.33	3309	0.17% ≤ CV ≤ 1.86%	0.89	22.89	1832	0.37% ≤ CV ≤ 3.18%	3.28	22.52	1130	0.22% ≤ CV ≤ 1.05%	0.40
	1000 nM	24.40	3156	0.25% ≤ CV ≤ 0.54%	0.30	23.04	1728	0.55% ≤ CV ≤ 2.43%	0.90	22.66	1052	0.17% ≤ CV ≤ 2.22%	2.01
**(b) HPV18**													
50 nM	400 nM	22.70	790	0.27% ≤ CV ≤ 1.14%	1.47	24.81	497	0.62% ≤ CV ≤ 2.91%	0.71	22.44	273	0.58% ≤ CV ≤ 2.11%	0.72
	600 nM	22.85	848	0.65% ≤ CV ≤ 1.13%	1.13	24.77	510	0.20% ≤ CV ≤ 2.29%	0.91	22.60	256	0.20% ≤ CV ≤ 1.89%	1.01
	800 nM	23.10	908	0.09% ≤ CV ≤ 0.47%	0.28	24.79	542	0.68% ≤ CV ≤ 2.14%	1.86	22.68	268	0.26% ≤ CV ≤ 1.28%	1.77
	1000 nM	23.26	835	0.24% ≤ CV ≤ 1.04%	1.44	24.75	507	0.60% ≤ CV ≤ 1.86%	1.36	22.78	237	0.26% ≤ CV ≤ 1.06%	1.36
100 nM	400 nM	22.33	1768	0.56% ≤ CV ≤ 1.74%	3.17	24.83	1025	0.44% ≤ CV ≤ 5.48%	3.13	22.93	610	0.09% ≤ CV ≤ 1.54%	2.22
	600 nM	22.79	1791	0.30% ≤ CV ≤ 0.62%	2.94	24.84	995	0.85% ≤ CV ≤ 3.24%	1.16	22.98	581	0.44% ≤ CV ≤ 1.24%	2.50
	800 nM	22.74	1877	0.60% ≤ CV ≤ 1.70%	3.73	24.69	1016	0.53% ≤ CV ≤ 0.69%	1.38	22.90	582	0.31% ≤ CV ≤ 1.70%	3.32
	1000 nM	22.97	1846	0.48% ≤ CV ≤ 1.54%	3.48	25.00	968	0.86% ≤ CV ≤ 2.23%	2.09	23.06	552	0.28% ≤ CV ≤ 1.51%	2.96
150 nM	400 nM	22.91	2115	0.39% ≤ CV ≤ 1.30%	1.06	24.81	1270	0.43% ≤ CV ≤ 2.61%	1.65	23.25	747	0.15% ≤ CV ≤ 2.11%	0.71
	600 nM	23.18	2313	0.26% ≤ CV ≤ 0.66%	1.07	25.50	1265	1.19% ≤ CV ≤ 7.30%	1.82	23.32	730	0.45% ≤ CV ≤ 1.00%	0.89
	800 nM	23.21	2366	0.29% ≤ CV ≤ 1.82%	0.56	24.90	1347	1.13% ≤ CV ≤ 3.23%	0.89	23.15	703	0.30% ≤ CV ≤ 1.37%	0.33
	1000 nM	23.36	2485	0.20% ≤ CV ≤ 1.35%	0.99	25.10	1384	0.62% ≤ CV ≤ 4.09%	0.43	23.35	725	0.85% ≤ CV ≤ 1.56%	0.70
200 nM	400 nM	23.01	2948	0.24% ≤ CV ≤ 1.09%	0.98	25.15	1687	1.34% ≤ CV ≤ 1.92%	0.83	23.36	1107	0.28% ≤ CV ≤ 0.64%	0.96
	600 nM	23.19	3298	0.33% ≤ CV ≤ 0.61%	0.64	25.45	1629	0.46% ≤ CV ≤ 2.32%	1.20	25.52	1075	0.20% ≤ CV ≤ 0.94%	1.15
	800 nM	23.33	3499	0.28% ≤ CV ≤ 0.60%	0.76	24.96	1734	0.36% ≤ CV ≤ 1.01%	1.78	23.49	1084	0.64% ≤ CV ≤ 0.88%	0.15
	1000 nM	23.51	3354	0.21% ≤ CV ≤ 1.16%	1.06	25.05	1702	0.98% ≤ CV ≤ 1.18%	1.69	23.65	1,000	0.14% ≤ CV ≤ 0.87%	0.58

*Ct* cycle threshold.

^a^Mean of inter-assay Ct from technical triplicate.

A temperature gradient (54 °C, 56.4 °C, 57.8 °C and 60 °C) was tested to select the best annealing temperature for the lowest Ct value (Table 3a,b). For the two multiplex qPCRs, Ct values were not significantly different among all the temperatures tested (p > 0.05). To be as specific as possible, we thus selected the annealing temperature of 60 °C as optimal temperature.

**Table 3:** Intra- and inter-assay reproducibility of HPV16 (a) and HPV18 (b) qPCR multiplexes for selected annealing temperatures.

	E6				E7				GAPDH			
	Coefficient of variation				Coefficient of variation				Coefficient of variation			
	Ct mean^a^	RFUs mean	Intra-assay	Inter-assay	Ct mean^a^	RFUs mean	Intra-assay	Inter-assay	Ct mean^a^	RFUs mean	Intra-assay	Inter-assay
**(a) HPV 16**												
Tm 60 °C	26.65	1999	0.61% ≤ CV ≤ 1.99%	2.12%	25.69	1674	0.46% ≤ CV ≤ 1.78%	2.67%	26.47	1065	0.53% ≤ CV ≤ 1.29%	0.46%
Tm 57.8 °C	26.66	1523	0.75% ≤ CV ≤ 3.03%	0.80%	25.02	1844	0.67% ≤ CV ≤ 2.85%	1.60%	26.60	1071	0.38% ≤ CV ≤ 0.85%	0.38%
Tm 56.4 °C	26.50	1230	0.91% ≤ CV ≤ 2.38%	1.42%	25.24	1854	1.66% ≤ CV ≤ 2.82%	2.09%	26.53	1115	0.47% ≤ CV ≤ 2.55%	0.72%
Tm 54 °C	26.60	1064	1.10% ≤ CV ≤ 3.17%	1.91%	25.43	1747	1.78% ≤ CV ≤ 3.45%	2.97%	26.58	1026	0.49% ≤ CV ≤ 2.05%	0.83%
**(b) HPV 18**												
Tm 60 °C	24.00	3180	0.07% ≤ CV ≤ 1.57%	3.12	26.63	1549	1.17% ≤ CV ≤ 2.92%	5.05%	24.59	1314	1.22% ≤ CV ≤ 3.12%	3.63%
Tm 57.8 °C	24.74	2488	0.47% ≤ CV ≤ 1.65%	1.76	27.19	1385	0.25% ≤ CV ≤ 2.03%	0.92%	25.08	1048	0.28% ≤ CV ≤ 1.22%	2.09%
Tm 56.4 °C	24.53	2613	0.44% ≤ CV ≤ 1.01%	1.80	27.13	1455	0.56% ≤ CV ≤ 1.85%	4.36%	24.97	1073	0.52% ≤ CV ≤ 1.16%	1.55%
Tm 54 °C	25.15	2760	0.82% ≤ CV ≤ 1.02%	4.56	27.31	1506	0.86% ≤ CV ≤ 2.05%	3.12%	25.92	1081	0.80% ≤ CV ≤ 2.03%	3.73%

*Ct* cycle threshold, *Cv* coefficient of variation.

^a^Mean of inter-assay Ct of technical triplicate.

### Multiplex qPCR specificity

**Figure 1:**
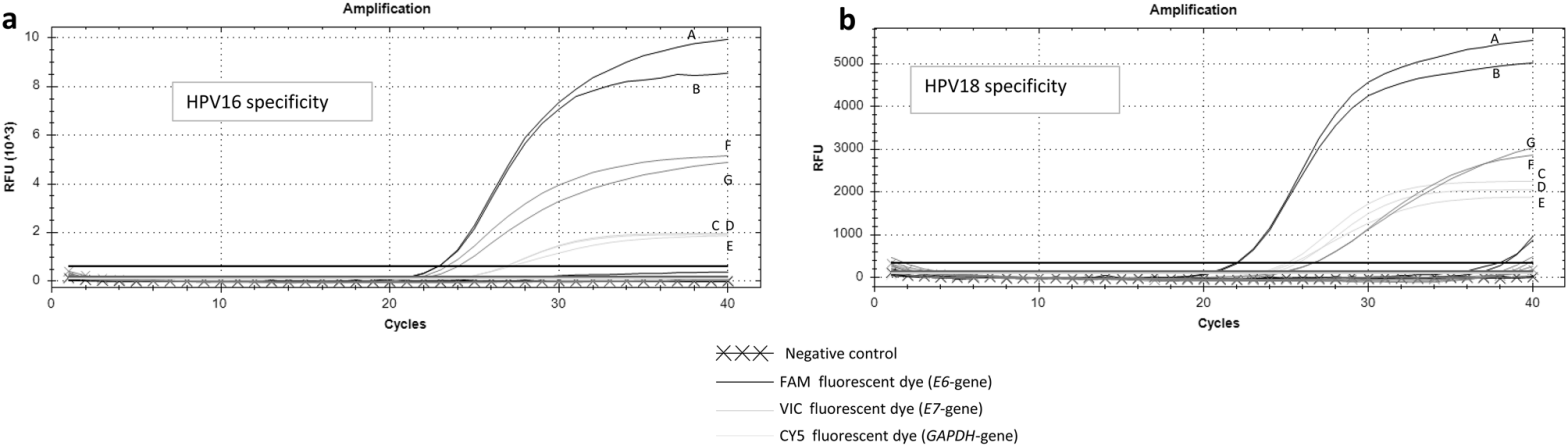
Amplification patterns obtained with the multiplex systems on plasmid DNA. The curves were generated by amplification of 10^5^ copies of plasmid/2µL of single or pooled *E6*, *E7* and/or GAPDH plasmid for HPV16 and HPV18. (**a**) Specificity of HPV16 *E6*, *E7* and *GAPDH* multiplex for the detection of HPV16 plasmid pool (A, E, G), HPV16 *E6* plasmid (B), *GAPDH* plasmid (C), HPV16 *E7* plasmid (F) and HPV18 plasmid pool (D) (**b**) Specificity of HPV18 *E6*, *E7* and *GAPDH* multiplex for the detection of HPV18 plasmid pool (B, E, G), HPV18 *E6* plasmid (A), *GAPDH* plasmid (C), HPV18 *E7* plasmid (F), HPV16 plasmid pool (D).

To determine the multiplex specificity for the two HPVs, each plasmid gene (*E6*, *E7*, *GAPDH*) separately or pooled were run in qPCR reaction mixes with the pool of primers and probes for HPV16 or HPV18 using the optimal qPCR parameters previously determined. qPCR resulted in a single curve for each specific plasmid run in the HPV16 multiplex mix. This indicated that this triplex was able to detect all the three genes without cross-amplification and non-specific background fluorescence (Fig. 1a). For HPV18, non-specific background amplification of *E6* and *E7* and cross-amplification between HPV16 and HPV18 were observed after a Ct of 38 (Fig. 1b).

### Sensitivity of the HPV multiplex qPCR

**Figure 2:**
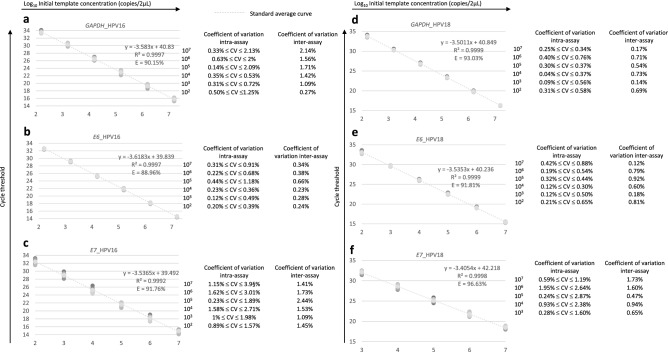
Sensibility and linearity of HPV16 and HPV18 triplexes. Standard curves were generated by amplification of serial dilutions from 10^7^ to 10^–2^ of *E6*, *E7* and *GAPDH* plasmid pool for HPV16 (**a**–**c**) and HPV18 (**d**–**f**) using multiplex qPCR. Each dilution was run in biological and technical triplicate. R^2^ represent the coefficient of determination and the PCR efficiency (E) of each target was calculated using the slope of each standard curve with the formula E = (10^–1/slope^ − 1)*100.

In the qPCR reactions, all standard curves showed a strong linear correlation (coefficient average R^2^ > 0.9990) for all tested genes (Fig. 2a–f). Amplification efficiencies for the HPV16 qPCR were 88.96% for *E6*, 91.76% for *E7* and 90.15% for *GAPDH* (Fig. 2a–c). Amplification efficiencies for the HPV18 qPCR were 91.81% for *E6*, 96.63% for *E7* and 93.03% for *GAPDH* (Fig. 2d–f). The regression curve slope was − 3.6183, − 3.5365 and − 3.583 for the *E6*, *E7* and *GAPDH* genes for the HPV16 triplex qPCR, respectively, and − 3.5353, − 3.4054 and − 3.5011 for the *E6*, *E7* and *GAPDH* genes of the HPV18 triplex qPCR, respectively.

Limits of detection (LOD) were determined as the last dilution of plasmids for which ≥ 95% of the intra- and inter-triplicates gave a positive result. The mean frequency of positives for the multiplex qPCR was 100% at 10^2^ plasmid copies/µL, and 97.88% and 97.34% at 10^1^ plasmid copies/µL for HPV16 and HPV18, respectively (Table 4).

**Table 4:** Positive frequency for multiplex with plasmid serial dilution (10^7^–10^0^ copies/µL). Positive frequency was calculated according to the ratio between the numbers of positive amplification detection and the number of expected amplification curve for each dilution of HPV16 (a) and HPV18 (b).

	10^0^ (%)	10^1^ (%)	10^2^ (%)	10^3^ (%)	10^4^ (%)	10^5^ (%)	10^6^ (%)	10^7^ (%)
**(a) HPV16 concentration (copy/µL)**								
*GAPDH*	83.3	93.65	100	100	100	100	100	100
*E6*	91.67	100	100	100	100	100	100	100
*E7*	91.67	100	100	100	100	100	100	100
Mean frequency of positive	88.88	97.88	100	100	100	100	100	100
**(b) HPV18 concentration (copy/µL)**								
*GAPDH*	91.67	98.4	100	100	100	100	100	100
*E6*	93.06	98.4	100	100	100	100	100	100
E7	83.33	95.24	100	100	100	100	100	100
Mean frequency of positive	89.35	97.34	100	100	100	100	100	100

### Assay reproducibility

The coefficient of variations for the biological (intra-assay) triplicates were all less than 8% and those for the technical (inter-assay) triplicates were all less than 5% (Tables 2 and 3).

### Assay performance of HPV16 and HPV18 multiplex qPCR

Concordance between results from our in-house multiplex qPCR systems and from the Xpert HPV Assay was analyzed for 96 clinical samples (Supplementary Table 1). Among these samples, 46 were detected positive for HPV16 and 38 for HPV18/45 (including three samples positive for both HPV16 and HPV18/45). Negative controls consisted in seven other HR-HPVs and eight HPV-negative samples (Supplementary Table 1).

Among the 46 samples found HPV16-positive by the Xpert HPV Assay, 44 were also positive using the triplex qPCR developed in this study. For these samples, Ct of the *GAPDH* gene ranged between 21.07 and 38.48 (median 24.32). The *E6* gene showed Ct values between 15.74 and 39.07 (median, 26.16) and the *E7* gene had a Ct range between 15.66 and 39.82 (median, 25.03). Seven samples presented Ct > 35 for *E6* or *E7* genes. For 3/7, only the *E6* gene was detected while for 1/7 only *E7* was detected. Co-infection with HPV18 was detected in 13 samples (e.g., samples no. 10, 31, 32, 36, 37, 40, 41, 43, 45, 47, 48, 64 and 90). For 2 samples (no. 47 and 48), the *GAPDH* gene was not detected whereas *E6* and *E7* oncogenes were early detected (Ct between 15.66 and 20.46). For one sample (no. 2), results were discordant between the Xpert HPV Assay and the multiplex qPCR developed here, as HPV18 was detected instead of HPV16.

Among the 38 HPV18/45 samples detected by the Xpert HPV Assay, 18 were found HPV18-positive using the triplex qPCR. Ct ranged between 21.19 and 30.45 (median, 24.66) for the *GAPDH* gene, between 15.18 and 38.90 (median, 30.32) for the *E6* gene and between 16.45 and 39.60 (median, 32.90) for the *E7* gene. Samples that were HPV18-negative were expected to be HPV45 and were verified by Sanger sequencing after conventional PCR using universal HPV primers^34^. The results confirmed HPV45 in seven cases, but also detected HPV53 in three cases. Co-infection with HPV16 was detected for three samples (no. 58, 64 and 90).

All negative samples (n = 8) were found negative, except one (sample no. 5), for which HPV16 and HPV18 co-infections were detected with late Ct values for *E7* gene, 38.02 and 36.67, respectively. HPV16 was also detected in one of the other HR-HPV samples (no. 24).

Considering the negative controls (negative samples and other HR-HPVs) and the positive samples, the concordance between the Xpert HPV Assay and the triplex qPCRs developed in this study was 93.44% for HPV16 with a Cohen’s kappa value of 0.82 (almost perfect agreement between the two tests) and 73.58% for HPV18 with a Cohen’s kappa value of 0.48 (moderate agreement).

In addition, for some clinical samples (e.g., samples no. 1, 3, 31, 39, 40, 43, 49, 58, 85), *E6* and *E7* oncogenes for both HPV18 and HPV16 were detected with at least 3 Ct earlier than the *GAPDH* gene (Supplementary Table 1 and Supplementary Fig. 1a,b).

Using the standard curves of each gene of interest, the viral load of HPV copies/10^6^ cells was determined (Supplementary Table 1). Viral load ranged from 3.52 × 10^1^ to 2.78 × 10^8^ HPV copies/10^6^ cells (± SD, 4.23 × 10^7^) for HPV16 and from 4.12 × 10^1^ to 7.87 × 10^8^ HPV copies/10^6^ (± SD, 1.48 × 10^8^) for HPV18. Some samples, particularly those from cervico-vaginal smears, presented a mean value close to the limit of detection previously defined at 10^1^ plasmid copy/µL (e.g., sample no. 29 and 41 for HPV16 and sample no. 90 for HPV18).

## Discussion

In most of cases (80–90%), HPV infections are transient as the result of viral clearance by the host immune system whereas viral persistence is necessary to induce HPV related-diseases (warts, cancers…)^35,36^. Between 60 and 90% of worldwide HPV cancers cases are due to HPV16 and/or HPV18^6^ and infection by one of those HPV types has a predictive value of cervical intraepithelial neoplasia grade 3 lesion^37^. The present study describes the development and validation of two real-time PCR triplexes for fast detection and relative quantification of *E6* and *E7* DNA oncogenes for HPV16 and HPV18.

Amplification efficiencies of each gene of the two multiplex systems ranged between 80 and 120% with good standard curve coefficient of correlation (R^2^ > 0.99). The difference between the PCR efficiencies obtained for each target gene of the multiplex system never exceeded 15%, which further demonstrated the absence of competition between the different targets^32,38^. The low coefficient of variation for both intra- (≤ 7.4%) and inter- (≤ 3.7%) assays showed a good reproducibility of each steps of the multiplex development. The triplex qPCR assays allowed the detection of up to 10^1^ plasmid copies/µL for all genes tested.

Currently, diagnosis of HPV infection often uses commercially available tests that target the complete genome, *L1* gene or *E6*/*E7* genes of a panel of HPV types (Hybrid Capture 2, Cervista HPV HR or HPV 16/18, Cobas HPV or Xpert HPV)^39^. Some of these qualitative HPV tests uses specific technologies such as hybrid capture with signal amplification by chemiluminescence (Hybrid Capture 2, Care HPV) or hybridization by FRET probe (Cervista HPV HR). Other HPV tests are easier to use but need specific additional laboratory equipment (Cobas HPV, Xpert HPV, Papillocheck). The two multiplex systems developed in this study allow detecting DNA of two of the most prevalent HR-HPVs and to quantify *E6* and *E7* oncogenes DNA levels compare to *GAPDH* housekeeping gene within the same amplification reaction. Moreover, in house probe-based real-time qPCR is a fast, easy to use, specific and reproducible method that only requires a minimal amount of DNA, does not need specific, assay-dedicated laboratory instrument, and can allow testing in the same run multiple sample DNA extracts on 96- or 384-well plates.

The multiplexes developed in this study confirmed the clinical diagnosis of the Xpert HPV assay with a concordance of 93.44% for HPV16 and 73.58% for HPV18. Lower concordance and Cohen’s kappa value were observed for HPV18 as the Xpert HPV assay provided a positive signal for both HPV18 and HPV45 whereas our system only targeted HPV18. Using HPV DNA quantification, we noted for some patients that oncogenes *E6* and *E7* have a Ct value lower than the *GAPDH* gene. This could be a signature of an active virus replication and transient infection. In contrast, patients with Ct for oncogenes equal to or higher than the *GAPDH* gene may reflect persistence under an episomal state or HPV integration to the human genome. In this work, 15 samples with HPV18 and HPV16 co-infections were detected. Detection of HPV co-infections is of particular interest as a combination of HPV16/18 co-infection has been associated with grade 2 and 3 cervical intraepithelial neoplasia (CIN)^40^. Viral loads per millions of human cells were determined for HPV16 and HPV18 as these could be a marker of CIN2 and be a predictor of lesion progression^27,41,42^. The value measured in this study were within the limits of the standard curves and showed large variations across patients and samples.

In 2010, a study showed that mRNA levels of *E6* and *E7* oncogenes are correlated with the severity of cervical intraepithelial neoplasia^28^. Quantification of mRNA could thus be a novel marker of HPV infection and cervical intraepithelial neoplasia progression^43^. So far, only few HPV detection test target mRNAs. These tests are qualitative (HPV-Proofer) and do not discriminate HPV types (Aptima HPV Assay). In 2018, a multiplex reverse transcription real-time PCR for *E6*/*E7* mRNA detection of 14 h-HPV without discrimination among types was developed^44^. This study confirmed the association of *E6*/*E7* mRNA increased levels with the severity of the cervical lesions. Another study showed that the detection of both *E6* and *E7* mRNAs showed significant association with the occurrence of upgraded abnormal cytology^45^. In this study, we could simultaneously detect and quantify *E6* and *E7* genes within the same amplification reaction. The triplex qPCR developed in this study could then be used to assess *E6*/*E7* oncogene mRNA levels for a personalized follow-up of patients with confirmed HPV18 and/or HPV16 persistent infections, in order to survey women at risk of developing cervical carcinoma.

To conclude, the multiplex PCR systems developed in this study could be used as extra support of traditional HPV tests to validate specifically the presence of and quantify per million cells HPV16 and/or HPV18 DNA. It may also be used for research in second intention for the follow-up of patients with confirmed infection who are at risk of developing lesions by quantifying expression levels of *E6* and *E7* oncogenes normalized on the *GAPDH* expression. This could be easily realized simultaneously on many samples (96–384 wells qPCR plate) and could be developed in the future for other less frequent HR-HPVs.

## Supplementary Information

Supplementary Legends.

Supplementary Table 1.

Supplementary Figure 1.
