# Supplementary material for: Development and validation of a multiplex qPCR assay for detection and relative quantification of HPV16 and HPV18 E6 and E7 oncogenes
Source: Sci Rep. 2021 Feb 17;11:4039. doi: 10.1038/s41598-021-83489-2 (PMC7889863; doi:10.1038/s41598-021-83489-2)
Supplement: Supplementary file 1 — Supplementary Legends. [file 41598_2021_83489_MOESM1_ESM.docx]

**Supplementary Table 1: Summary of HPV16/HPV18 DNA detection screened by multiplex qPCR sytems.** Nature of the samples, diagnostic results from the IHU Méditerranée Infection laboratory, HPV16 and HPV18 *E6*, *E7* and *GAPDH* molecular screening by qPCR using the multiplexes developed in this study, mean HPV16 and HPV18 viral load. Ct, Cycle threshold; ND not detected; NA not applicable (could not be calculated as no GAPDH Ct was detected).

**Supplementary Figure 1: Examples of early detection of *E6* and *E7* oncogenes for HP16 and HPV18 triplexes (a)** Amplification pattern obtain with HPV16 triplex with Ct values of *E6* and *E7* oncogenes higher than *GAPDH* **(b)** Amplification pattern obtain with HPV18 triplex with Ct values of *E6* and *E7* oncogenes higher than *GAPDH*
