# Supplementary material for: Development and validation of a multiplex qPCR assay for detection and relative quantification of HPV16 and HPV18 E6 and E7 oncogenes
Source: Sci Rep. 2021 Feb 17;11:4039. doi: 10.1038/s41598-021-83489-2 (PMC7889863; doi:10.1038/s41598-021-83489-2)
Supplement: Supplementary file 2 — Supplementary Table 1. [file 41598_2021_83489_MOESM2_ESM.pdf]

Supplementary Table 1

				HPV16 cycle threshold			HPV18 cycle threshold				Mean viral load of HPV (HPV copies/10^6 cells)	
Sample number	Sample nature	Xpert HPV assay results	Triplex results	GAPDH	E6	E7	GAPDH	E6	E7	Sanger sequencing results	HPV16	HPV18
1	Rectal swab	HPV18 or 45	HPV18	30.52	ND	ND	30.45	22.14	22.57	-	ND	7,87x10^{8}
2	Cervico-vaginal smear	HPV16	HPV18	29.21	ND	ND	28.54	ND	38.84	-	ND	6,14x10^{3}
3	Cervico-vaginal smear	HPV16	HPV16	29.26	26.09	20.9	27.96	ND	ND	-	7,21x10^{7}	ND
4	Anal smear	HPV18 or 45	HPV18	29.52	ND	ND	28.86	ND	39.6	-	ND	4,54x10^{3}
5	Anal smear	-	HPV16 + HPV18	32.63	ND	38.02	32.35	ND	36.67	-	1,73x10^{4}	3,26x10^{5}
6	Rectal swab	HPV18 or 45	-	31.24	ND	ND	32.02	ND	ND	HPV 53	ND	ND
7	Cervico-vaginal smear	HPV16	HPV16	32.65	34.24	31.54	33.34	ND	ND	-	7,66x10^{5}	ND
8	Cervico-vaginal smear	-	-	32.45	ND	ND	32.15	ND	ND	-	ND	ND
9	Rectal swab	HPV18 or 45	HPV18	27.49	ND	ND	28.02	36.27	38.31	-	ND	5,99x10^{3}
10	Cervix	HPV16	HPV16 + HPV18	28.22	38.35	ND	28.54	ND	38.31	-	1,46x10^{3}	8,79x10^{3}
11	Anal smear	HPV16	HPV16	38.48	37.23	37.97	ND	ND	ND	-	1,47x10^{6}	ND
12	Rectal swab	HPV18 or 45	-	36.08	ND	ND	37.19	ND	ND	-	ND	ND
13	Anal smear	HR-HPV other(s)	-	31.14	ND	ND	31.47	ND	ND	-	ND	ND
14	Anal smear	HPV16	HPV16	31.72	30.66	30.25	31.07	ND	ND	-	1,68x10^{6}	ND
15	Anal smear	-	-	26.36	ND	ND	26.24	ND	ND	-	ND	ND
16	Anal smear	-	-	28.9	ND	ND	29.02	ND	ND	-	ND	ND
17	Anal smear	-	-	26.69	ND	ND	26.72	ND	ND	-	ND	ND
18	Anal smear	HR-HPV other(s)	-	26.18	ND	ND	26.15	ND	ND	-	ND	ND
19	Anal smear	HPV16	HPV16	28.69	27.23	26.14	28.84	ND	ND	-	2,74x10^{6}	ND
20	Anal smear	HR-HPV other(s)	-	26.44	ND	ND	26.3	ND	ND	-	ND	ND
21	Anal smear	-	-	27.42	ND	ND	28.12	ND	ND	-	ND	ND
22	Anal smear	-	-	23.73	ND	ND	24.24	ND	ND	-	ND	ND
23	Anal smear	HR-HPV other(s)	-	29.35	ND	ND	30.07	ND	ND	-	ND	ND
24	Anal smear	HR-HPV other(s)	HPV16	25.11	33.68	36.07	26.43	ND	ND	-	2,17x10^{3}	ND
25	Anal smear	HR-HPV other(s)	-	34.39	ND	ND	35.07	ND	ND	-	ND	ND
26	Anal smear	HPV16	HPV16	24.02	23.35	22.61	23.59	ND	ND	-	1,46x10^{6}	ND
27	Anal smear	-	-	28.07	ND	ND	28.63	ND	ND	-	ND	ND
28	Anal smear	HR-HPV other(s)	-	37.22	ND	ND	38.46	ND	ND	-	ND	ND
29	Cervico-vaginal smear	HPV16	HPV16	23.3	39.07	ND	24.64	ND	ND	-	3,91x10^{1}	ND
30	Cervico-vaginal smear	HPV16	HPV16	21.42	24.26	24.26	23.11	ND	ND	-	1,22x10^{5}	ND
31	Cervico-vaginal smear	HPV16	HPV16 + HPV18	26.08	22.38	22.01	27.87	ND	37.9	-	9,09x10^{6}	7,47x10^{3}
32	Cervico-vaginal smear	HPV16	HPV16 + HPV18	23.25	22.33	22.19	27.69	ND	38.6	-	1,42x10^{6}	4,13x10^{3}
33	Cervico-vaginal smear	HPV16	HPV16	26.83	ND	39.82	24.62	ND	ND	-	1,29x10^{2}	ND
34	Cervico-vaginal smear	HPV16	HPV16	23.13	27.94	27.07	24.48	ND	ND	-	4,49x10^{4}	ND
35	Cervico-vaginal smear	HPV16	HPV16	23.14	31.93	32.6	24.48	ND	ND	-	2,32x10^{3}	ND
36	Cervico-vaginal smear	HPV16	HPV16 + HPV18	25.23	24.18	24.13	26.47	ND	38.41	-	1,51x10^{6}	2,11x10^{3}
37	Cervico-vaginal smear	HPV16	HPV16 + HPV18	29.13	19.08	28.46	31.02	ND	37.8	-	2,78x10^{8}	6,34x10^{4}
38	Cervico-vaginal smear	HPV16	HPV16	25.57	27.46	25.03	27	ND	ND	-	5,71x10^{5}	ND
39	Cervico-vaginal smear	HPV16	HPV16	23.29	18.46	18.82	24.42	ND	ND	-	1,54x10^{7}	ND
40	Cervico-vaginal smear	HPV16	HPV16 + HPV18	23.89	18.6	18.95	24.82	ND	38.9	-	2,07x10^{7}	5,11x10^{2}
41	Cervico-vaginal smear	HPV16	HPV16 + HPV18	22.88	38.81	ND	24.58	38.25	36.14	-	3,52x10^{1}	1,49x10^{3}
42	Cervico-vaginal smear	HPV16	HPV16	24.87	23.83	21.39	26.25	ND	ND	-	3,84x10^{6}	ND
43	Cervico-vaginal smear	HPV16	HPV16 + HPV18	28.91	24.67	24.89	31.06	ND	34.44	-	1,09x10^{7}	6,31x10^{5}
44	Cervico-vaginal smear	HPV16	HPV16	24.68	23.03	19.36	25.89	ND	ND	-	1,11x10^{7}	ND
45	Cervico-vaginal smear	HPV16	HPV16 + HPV18	31.53	32.07	31.09	33.23	ND	39.63	-	7,21x10^{5}	7,87x10^{4}
46	Cervico-vaginal smear	HPV16	HPV16	23.13	29.6	19.64	24.72	ND	ND	-	3,04x10^{6}	ND
47	Cervico-vaginal smear	HPV16	HPV16 + HPV18	ND	20.46	20.07	29.34	ND	37.73	-	NA	2,20x10^{4}
48	Cervico-vaginal smear	HPV16	HPV16 + HPV18	ND	15.74	15.66	28.07	ND	37.07	-	NA	1,49x10^{4}
49	Cervico-vaginal smear	HPV18 or 45	HPV18	25.85	ND	ND	25.49	21.57	22.44	-	ND	3,49x10^{7}
50	Cervico-vaginal smear	HPV18 or 45	HPV18	21.03	ND	ND	21.19	26.51	32.76	-	ND	2,00x10^{4}
51	Cervico-vaginal smear	HPV18 or 45	-	25.2	ND	ND	24.67	ND	ND	HPV 53	ND	ND
52	Cervico-vaginal smear	HPV18 or 45	-	25.24	ND	ND	24.73	ND	ND	HPV 45	ND	ND
53	Cervico-vaginal smear	HPV18 or 45	-	24.63	ND	ND	24.3	ND	ND	HPV 45	ND	ND
54	Cervico-vaginal smear	HPV18 or 45	HPV18	24.43	ND	ND	24.08	31.35	37.35	-	ND	5,74x10^{3}
55	Cervico-vaginal smear	HPV18 or 45	HPV18	24.82	ND	ND	24.4	ND	36.95	-	ND	1,45x10^{3}
56	Cervico-vaginal smear	HPV18 or 45	-	25.1	ND	ND	24.35	ND	ND	HPV 45	ND	ND
57	Cervico-vaginal smear	HPV18 or 45	HPV18	24.68	ND	ND	24.36	29.61	37.16	-	ND	2,04x10^{4}
58	Cervico-vaginal smear	HPV18 or 45	HPV16 + HPV18	21.71	30.85	29.96	22.16	15.18	16.45	-	2,78x10^{3}	2,30x10^{8}
59	Cervico-vaginal smear	HPV18 or 45	HPV18	23.26	ND	ND	22.89	29.02	33.15	-	ND	1,45x10^{4}
60	Cervico-vaginal smear	HPV18 or 45	HPV18	26.88	ND	ND	26.37	31.02	33.04	-	ND	6,68x10^{4}
61	Cervico-vaginal smear	HPV18 or 45	-	25.33	ND	ND	24.98	ND	ND	HPV 45	ND	ND
62	Cervico-vaginal smear	HPV18 or 45	-	24.88	ND	ND	24.52	ND	ND	HPV 45	ND	ND
63	Cervico-vaginal smear	HPV18 or 45	HPV18	25.17	ND	ND	24.72	38.32	ND	-	ND	1,72x10^{2}
64	Cervico-vaginal smear	HPV16 + HPV18 or 45	HPV16 + HPV18	24.21	29.85	26.71	23.92	35.94	ND	-	7,32x10^{4}	4,80x10^{2}
65	Cervico-vaginal smear	HPV18 or 45	HPV18	26.87	ND	ND	26.5	38.9	ND	-	ND	3,81x10^{2}
66	Cervico-vaginal smear	HPV18 or 45	-	21.86	ND	ND	21.37	ND	ND	HPV 45	ND	ND
67	Cervico-vaginal smear	HPV18 or 45	-	24.64	ND	ND	24.18	ND	ND	HPV 45	ND	ND
68	Vaginal swab	HPV18 or 45	-	23.07	ND	ND	22.84	ND	ND	HPV 53	ND	ND
69	Cervico-vaginal smear	HPV16	HPV16	23.51	28.8	28.17	23.42	ND	ND	-	3.04x10^{4}	ND
70	Cervico-vaginal smear	HPV18 or 45	-	22.61	ND	ND	22.3	ND	ND	-	ND	ND
71	Cervico-vaginal smear	HPV16	HPV16	22.39	19.57	19.19	21.9	ND	ND	-	5.19x10^{6}	ND
72	Vaginal swab	HPV18 or 45	HPV18	24.65	ND	ND	26.05	31.76	31.41	-	ND	1.06x10^{5}
73	Cervico-vaginal smear	HPV18 or 45	HPV18	24.36	ND	ND	24.6	20.75	21.32	-	ND	3.95x10^{7}
74	Cervico-vaginal smear	HPV16	-	23.36	ND	ND	23.19	ND	ND	-	ND	ND
75	Cervico-vaginal smear	HPV18 or 45	-	23.62	ND	ND	23.27	ND	ND	-	ND	ND
76	Cervico-vaginal smear	HPV16	HPV16	23.92	22.4	21.91	23.7	ND	ND	-	2.33x10^{6}	ND
77	Cervico-vaginal smear	HPV16	HPV16	23.57	25.97	25.68	23.33	ND	ND	-	1.76x10^{5}	ND
78	Cervico-vaginal smear	HPV16	HPV16	24.43	35.56	37.36	24.13	ND	ND	-	4.45x10^{2}	ND
79	Cervico-vaginal smear	HPV16	HPV16	24.19	26.85	24.87	25.26	ND	ND	-	2.81x10^{5}	ND
80	Cervico-vaginal smear	HPV18 or 45	-	21.83	ND	ND	22.02	ND	ND	-	ND	ND
81	Cervico-vaginal smear	HPV18 or 45	HPV18	26.26	ND	ND	27.14	21.59	21.5	-	ND	1.74x10^{8}
82	Cervico-vaginal smear	HPV16	HPV16	25.47	26.16	26.72	25.39	ND	ND	-	4.27x10^{5}	ND
83	Cervico-vaginal smear	HPV16	HPV16	25.59	25.33	25.35	25.62	ND	ND	-	8.91x10^{5}	ND
84	Cervico-vaginal smear	HPV16	HPV16	25.31	26.52	26.09	25.33	ND	ND	-	3.94x10^{5}	ND
85	Cervico-vaginal smear	HPV16	HPV16	23.24	19.41	18.4	22.61	ND	ND	-	1.24x10^{7}	ND
86	Cervico-vaginal smear	HPV18 or 45	-	24.01	ND	ND	23.71	ND	ND	-	ND	ND
87	Cervico-vaginal smear	HPV16	HPV16	21.07	22.28	22.55	21.15	ND	ND	-	3.25x10^{5}	ND
88	Cervico-vaginal smear	HPV18 or 45	-	23.02	ND	ND	22.43	ND	ND	-	ND	ND
89	Rectal swab	HPV18 or 45	-	29.86	ND	ND	29.73	ND	ND	-	ND	ND
90	Cervico-vaginal smear	HPV16 + HPV18 or 45	HPV16 + HPV18	23.4	30.04	29.37	23.02	38.8	NA	-	1.29x10^{4}	4.12x10^{1}
91	Cervico-vaginal smear	HPV16	HPV16	21.91	29.65	28.91	21.44	ND	ND	-	6.52x10^{3}	ND
92	Vaginal swab	HPV16	HPV16	24.72	22.25	21.44	25.01	ND	ND	-	4.77x10^{6}	ND
93	Vaginal swab	HPV16	HPV16	24.81	27.74	27.28	24.66	ND	ND	-	1.31x10^{5}	ND
94	Cervico-vaginal smear	HPV18 or 45	-	24.56	NA	NA	24.18	ND	ND	-	ND	ND
95	Cervico-vaginal smear	HPV16 + HPV18 or 45	HPV16	23.85	35.52	37.88	24.15	ND	ND	-	3x10^{2}	ND
96	Cervico-vaginal smear	HPV18 or 45	-	23.34	ND	ND	23.48	ND	ND	-	ND	ND
