# Supplementary material for: Development and validation of a multiplex qPCR assay for detection and relative quantification of HPV16 and HPV18 E6 and E7 oncogenes
Source: Sci Rep. 2021 Feb 17;11:4039. doi: 10.1038/s41598-021-83489-2 (PMC7889863; doi:10.1038/s41598-021-83489-2)

Development and validation of a multiplex qPCR assay for detection and relative quantification of HPV16 and HPV18 *E6* and *E7* oncogenes

Alexia Bordigoni, Anne Motte, Hervé Tissot-Dupont, Philippe Colson, and Christelle Desnues

Supplementary Figure 1

a

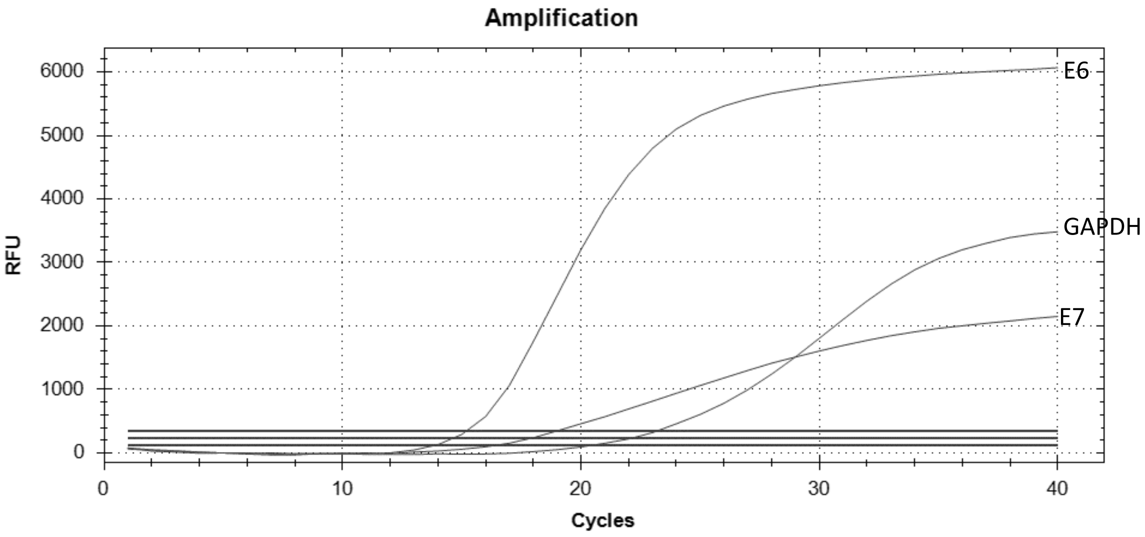

b

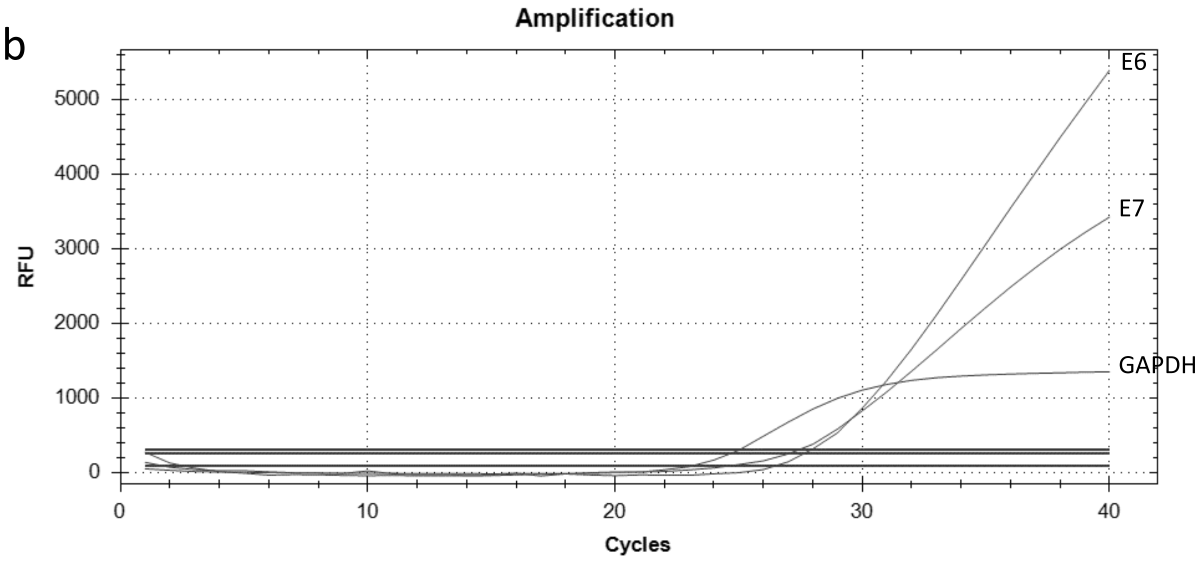

Supplement: Supplementary file 3 — Supplementary Figure 1. [file 41598_2021_83489_MOESM3_ESM.pdf]
